# Expression of NF-κB p50 in Tumor Stroma Limits the Control of Tumors by Radiation Therapy

**DOI:** 10.1371/journal.pone.0039295

**Published:** 2012-06-28

**Authors:** Marka R. Crittenden, Benjamin Cottam, Talicia Savage, Cynthia Nguyen, Pippa Newell, Michael J. Gough

**Affiliations:** 1 Earle A. Chiles Research Institute, Providence Portland Medical Center, Portland, Oregon, United States of America; 2 Providence Hepatobiliary and Pancreatic Cancer Program, Providence Portland Medical Center, Portland, Oregon, United States of America; 3 The Oregon Clinic, Portland, Oregon, United States of America; City of Hope National Medical Center and Beckman Research Institute, United States of America

## Abstract

Radiation therapy aims to kill cancer cells with a minimum of normal tissue toxicity. Dying cancer cells have been proposed to be a source of tumor antigens and may release endogenous immune adjuvants into the tumor environment. For these reasons, radiation therapy may be an effective modality to initiate new anti-tumor adaptive immune responses that can target residual disease and distant metastases. However, tumors engender an environment dominated by M2 differentiated tumor macrophages that support tumor invasion, metastases and escape from immune control. In this study, we demonstrate that following radiation therapy of tumors in mice, there is an influx of tumor macrophages that ultimately polarize towards immune suppression. We demonstrate using *in vitro* models that this polarization is mediated by transcriptional regulation by NFκB p50, and that in mice lacking NFκB p50, radiation therapy is more effective. We propose that despite the opportunity for increased antigen-specific adaptive immune responses, the intrinsic processes of repair following radiation therapy may limit the ability to control residual disease.

## Introduction

There exists an array of cytotoxic therapies that can dramatically reduce the tumor to only a few cells with clonogenic potential. Unfortunately for cancer patients, tumors can recur from these small pockets of residual disease. The emergence of metastases from residual microscopic disease is a major source of mortality in cancer patients. In animal models of cancer therapy, it is becoming clear that immune responses play a role in the success of cytotoxic therapies [1], [2], [3], and that the outcome following cytotoxic therapies can be improved by enhancing adaptive immune responses [1], [3], [4]. By causing the death of cancer cells, radiation therapy has been proposed to provide both tumor antigen and endogenous immune adjuvants to initiate *de novo* tumor-specific immune responses.

The majority of cytotoxic cancer therapies result in cancer cell death through the induction of apoptosis. If the phagocytic capacity of tumor macrophages is overwhelmed, apoptotic cells can progress to secondary necrosis and result in induction of pro-inflammatory cytokines from macrophages [5]. Catastrophic death of cancer cells can result in release of endogenous immune adjuvants that can alter immune responses, such as heat shock proteins, calreticulin and HMGB1 [2], [6]. In addition, a range of studies have demonstrated that it may be possible to select a cytotoxic agent that promotes immunogenic, non-apoptotic cell death, or redirects the mechanism of cell death [2], [7]. Studies demonstrate that expression of TLR4, a key receptor for immunological adjuvants, is critical both for vaccination with tumor cells killed via radiation or chemotherapy, and the efficacy of cytotoxic therapy *in vivo* [8]. These data fit a model where adjuvants released from dying cancer cells may play a role in establishing functional anti-tumor immune responses.

Despite the potential immunogenicity of endogenous adjuvants, the outcome of adjuvant release is heavily influenced by the differentiation of cells in the environment. Alternative (M2) activation of macrophages results in a distinct response to adjuvant compared to classically activated (M1) macrophages. M1 macrophages respond to adjuvant with secretion of pro-inflammatory cytokines such as TNFα, whereas M2 macrophages respond to adjuvant with secretion of anti-inflammatory cytokines such as IL-10 [9]. Exposure to apoptotic cells cause macrophages to secrete a range of anti-inflammatory cytokines, including IL-10 and TGF-β [5], [7], [10]. Thus, radiation-induced death of cancer cells may result in M2 activation via the effect of apoptotic cells, such that any subsequent release of adjuvants from cancer cells undergoing secondary necrosis will cause immune suppression. The suppressive response of M2 macrophages is a key feature of inflammatory resolution, which serves to repair inflammatory destruction following control of infections by laying down supportive matrix, establishing vascular structures, and terminating adaptive immune responses [11], [12]. Importantly, these functions are commonly observed in tumor macrophages, which drive angiogenesis and adaptive immune suppression in the tumor [13], [14], [15], [16].

For these reasons it is critical to understand the tumor environment during cytotoxic therapies to optimize the contribution of immune cells to cancer control. We demonstrate that established tumors display a limited response to radiation therapy and that treatment is followed by a significant influx of M2-differentiated macrophages into the tumor stroma. We demonstrate that NFκB p50 provides a transcriptional mechanism for polarization of macrophages cells in the presence of irradiated cancer cells. We demonstrate that *in vivo* radiation therapy is more effective in mice defective in M2 polarization though deletion of NFκB p50. We propose that the polarization of tumor macrophages is a limitation for adaptive immune control of residual disease and may be a target to enhance the efficacy of cytotoxic therapies.

## Materials and Methods

### Animals and Cell Lines

The Raw264.7 monocyte/macrophage cell line [17] and the 4T1 mammary carcinoma cell line [18] were obtained from the ATCC (Manassas, VA). The Panc02 murine pancreatic adenocarcinoma cell line [19], (C57BL/6) was kindly provided by Dr Woo (Mount Sinai School of Medicine, NY). 6–8 week old C57BL/6 mice and Balb/c were obtained from Charles River Laboratories (Wilmington, MA) for use in these experiments. NFκB1^−/−^ mice were obtained from The Jackson Laboratory (Bar Harbor, ME). All animal protocols were approved by the Earle A. Chiles Research Institute IACUC (Animal Welfare Assurance No. A3913-01).

### Antibodies and Reagents

The FACS antibodies CD11b, Gr1 and IA (MHC class II) were purchased from Ebioscience (San Diego, CA). Ultrapure LPS was purchased from Invivogen (San Diego, CA). Western blotting antibodies used include Arginase I (BD biosciences, San Jose, CA), iNOS (Cayman Chemical Corporation, Ann Arbor, MI), GAPdH, anti-mouse-HRP, and anti-rabbit-HRP (all Cell Signaling Technology, Danvers, MA). Rat anti-F4/80 was purchased from AbD Serotec (Raleigh, NC), rabbit anti-Von Willebrand Factor (VWF) was purchased from Abcam, and the secondary antibodies were anti-Rabbit Alexa Fluor 488 and anti-Rat Alexa Fluor 568 (Invitrogen).

### Radiation Therapy of Tumors

Tumors were innoculated s.c. in the right leg below the knee at a dose of 2×10^5^ Panc02 or 5×10^4^ 4T1 cells and allowed to establish for 14–17 days before initiation of treatment. Three daily 20 Gy treatment fractions were given using an Elekta Synergy linear accelerator (Atlanta, GA) with 6 MV photons with 1 cm bolus and incorporating a half beam block to minimize dose to the torso.

### Isolation and Analysis of Cancer Cells and Tumor Infiltrating Cells

For clonogenic analysis of cancer cells, the tumor was dissected into approximately 2 mm fragments followed by agitation in 1 mg/mL collagenase (Invitrogen), 100 µg/mL hyaluronidase (Sigma), and 20 mg/mL DNase (Sigma) in PBS for 1 hr at room temperature. The digest was filtered through 100 µm nylon mesh to remove macroscopic debris. Serial dilutions of tumor cells were seeded to 6-well tissue culture plates in media containing 60 µM 6-thioguanine and colonies were counted after 7 days. The serial dilution and the colony number were used to calculate the number of clonogenic cancer cells in the original tumor. For FACS analysis of tumor infiltrating immune cells, cells suspensions were stained with antibodies specific for CD11b, IA (MHC class II) and Gr1 as previously described [20]. The proportion of each infiltrating cell type was analyzed on a BD LSRII. FACS sorting of tumor macrophages was performed as previously described [20] using a BD FACSAria Cell Sorter to greater than 98% purity. The morphology of the sorted cell populations was determined by cytospin followed by DiffQuick staining.

### Immunohistology

Tumors were fixed in formalin overnight, cyropreserved by equilibration in 30% sucrose then flash frozen in OCT. 10 µM cryosections were cut and every 5^th^ section was H&E stained for orientation. Neighboring sections were stained with primary antibodies specific for F4/80 and Von Willebrand Factor (VWF) and binding detected with Alexa Fluor 488 or Alexa Fluor 568 conjugated secondary antibodies, respectively. Sections were mounted in the presence of DAPI (Invitrogen, Carlsbad, CA) to stain nuclear material. Images were acquired using a Zeiss LSM 510-meta confocal microscope at the Oregon Medical Laser Center.

### Gene Expression Microarrays

Total RNA was prepared from FACS sorted CD11b^+^IA^+^ cells using a PrepEase RNA Spin Kit (Affymetrix, Cleveland, OH). The Affymetrix Microarray Core Facility at Oregon Health and Sciences University (Portland, OR) prepared DNA probes and performed Microarray Analysis. Gene expression data has been uploaded to GEO (Accession number GSE34206). Data was analyzed using GeneSifter (Geospiza Inc, Seattle, WA).

### Western Blotting

Cells were lysed in RIPA buffer and denatured in SDS loading buffer containing β2-mercaptoethanol, electrophoresed on 10% SDS-PAGE gels and transferred to nitrocellulose. Blocked blots were probed overnight at 4°C with primary antibodies followed by HRP-conjugated secondary antibodies. Binding was detected using a Pierce SuperSignal Pico Chemiluminescent Substrate (Thermo Fisher Scientific, Rockford, IL) and exposure to film.

### Preparation of Bone Marrow Macrophages

Bone marrow cells isolated from long-bones of mice were cultured for a total of 7 days in complete media containing 40 ng/ml MCSF (Ebioscience), with additional growth media provided after 3 days of culture. Adherent cells were harvested and macrophage differentiation confirmed by flow cytometry for CD11b, F4/80, Gr1 and IA.

### Cancer Cell-macrophage Co-cultures

Panc02 or 4T1 cells were irradiated with a 10 Gy dose using a cesium source, and 1×10^4^ cancer cells were co-cultured with 1×10^4^ Raw264.7 cells or 2×10^4^ primary bone marrow macrophages in replicate wells of 96-well u-bottomed plates for 24 hours before treatment with 100 ng/ml LPS. Supernatants were collected after a further 48 hours and tested for cytokine levels by ELISA using matched antibody pairs specific for TNF and IL-10 (R&D Systems, Minneapolis, MN) against a standard curve of recombinant cytokine.

### Statistics

Kaplan and Meier survival curves were compared using a log-rank test. The gene expression phenotypes in each group were compared using analysis of variance (ANOVA). The difference in cytokine levels between specific groups was compared using Student’s T-test.

## Results

**Figure 1:**
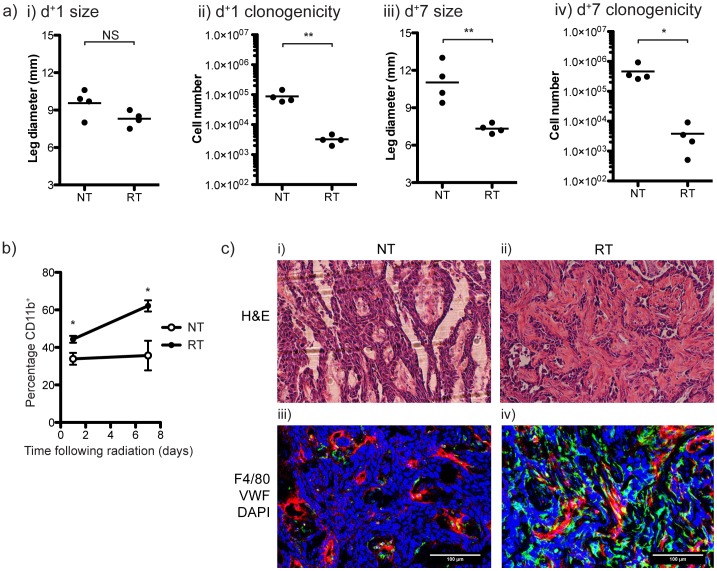
Radiation therapy of tumors. a) C57BL/6 mice were challenged with 2×10^5^ Panc02 *s.c.* in the right leg and mice received 3 daily doses of 20 Gy focal radiation to the leg beginning on day 14 (RT) or were left untreated (NT). i-ii) 1 day or iii-iv) 7 days following the final radiation dose, tumors were harvested, digested and clonogenic assays performed. b) One and seven days following the final radiation dose, tumors were harvested and tumor-infiltrating cells determined by FACS analysis. Graphs show the mean and standard error of tumor infiltrating CD11b^+^ cells in Panc02 tumors, and include data from two replicate experiments. c) Panc02 tumors were harvested for histology 7 days following the final radiation dose. Images show representative regions of neighboring sections from tumors receiving NT (i & iii) or RT (ii & iv) that were i-ii) H&E stained or iii-iv) underwent immunofluorescence staining with antibodies specific for VWF and F4/80, and detected with antibodies conjugated to AF488 (Green) and AF568 (Red), respectively. Nuclear material was counterstained with DAPI (Blue) and sections were imaged by confocal microscopy.

To investigate the tumor immune environment following radiation therapy, we developed a model of *in vivo* radiation therapy in immunocompetent mice. C57BL/6 mice challenged s.c. in the leg with Panc02 pancreatic adenocarcinoma were allowed to establish for 14 days. Mice were then treated with 20 Gy ×3 focal radiation to the leg over 3 days. Treatment of the mouse with the leg extended encompassed the tumor, but the half beam block ensured rapid dose drop-off and the treatment field did not include the tumor draining subiliac lymph node. In this model, radiation therapy had a statistically significant, but limited effect on the growth of the tumor and on the survival of tumor-bearing mice. To investigate the efficacy of radiation therapy on cancer cells, tumors were harvested from mice bearing Panc02 tumors 1 day or 7 days following the final dose of radiation and cancer cell viability determined by clonogenic assay (Figure 1A). There was a significant decrease in the clonogenic capacity of the cancer cells 1 day following the final radiation dose that was sustained at seven days, while the untreated tumors continued to grow. These data suggest that the eventual tumor progression results from an aggressive outgrowth of these residual viable cancer cells within the post-radiation environment. Macrophages are a significant component of the tumor environment, and have an important role in the response to dying cells [5], [7], [10] and in directing the repair of damaged tissues [11], [12]. To examine the consequence of radiation-induced cancer cell death on tumor macrophages, we harvested tumors from mice bearing Panc02 tumors 1 day or 7 days following the final dose of radiation and performed flow cytometry on tumor-infiltrating cells. The proportion of CD11b^+^ cells is significantly increased in tumors 1 day following the final radiation dose (Figure 1B) and further increased at 7 days following radiation. This increase in CD11b^+^ cells represents infiltration of multiple myeloid populations into the tumor. To assess macrophage infiltration, Panc02 tumors were harvested 7 days following the last dose of radiation and sections were stained for F4/80^+^ macrophages. F4/80^+^ macrophages were found in the stromal area of the untreated tumor and predominantly clustered around vascular structures (Figure 1C), though patchy areas of increased macrophage infiltration were visible when examining the entire untreated tumor (Figure S1). Following radiation there is an expansion in the stromal area readily detectible in the H&E staining (Figure 1C, image i-ii), associated with an increase in F4/80^+^ macrophages throughout the stroma (Figure 1C, image iii-iv). Analysis of the entire tumor demonstrates that this macrophage infiltration occurs throughout the treated tumor (Figure S1). These data demonstrate that there is a significant increase in macrophages in the tumor in response to radiation therapy and that these macrophages infiltrate into tumor stroma.

**Figure 2:**
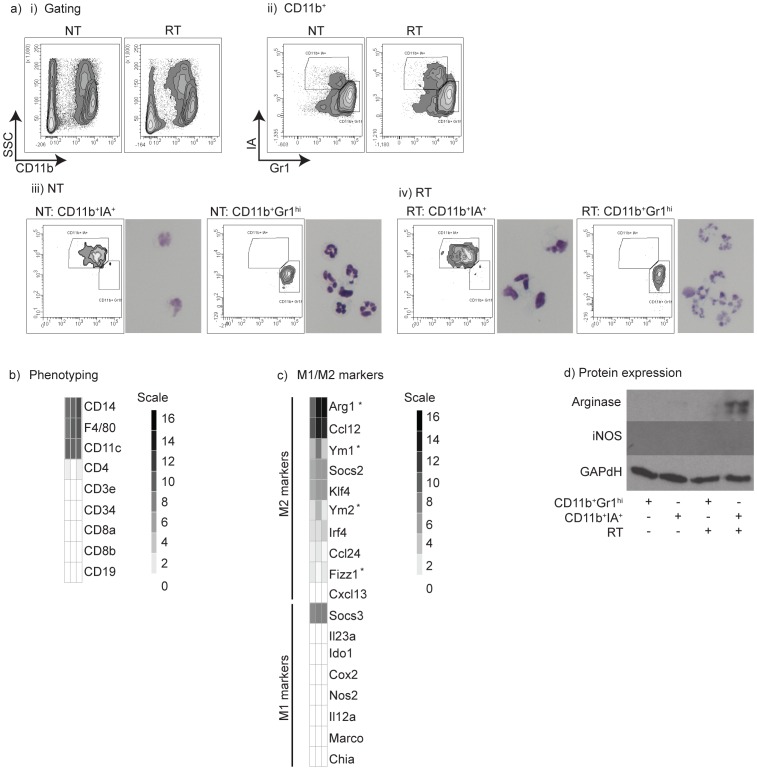
Gene expression microarray of tumor macrophages following radiation. a) Panc02 tumors were harvested 1 and 7 days following the final radiation dose and i) gated CD11b^+^ cells were FACS sorted according to expression of ii) Gr1 and IA. Sorted populations of CD11b^+^Gr1^hi^ and CD11b^+^IA^+^ cells from iii) NT or iv) RT tumors were tested for morphology by cytopsin and Diff-Quik staining. RNA was prepared from sorted CD11b^+^IA^+^ cells and Affymetrix gene expression microarray analysis was performed. Gene expression profiles were analyzed for the expression of b) lineage markers and c) M1 and M2-associated markers. d) CD11b^+^Gr1^hi^ and CD11b^+^Gr1^lo^ cells were sorted as in a) and lysates prepared from sorted cells for western blotting. The image represents 3 western blots cropped and positioned above each other to show detection of Arginase I, iNos and GAPDH. Gene array analysis uses RNA collected from purified macrophages isolated in 2 replicate experiments. Each gene list is sorted by gene expression level and includes an individual key showing the gene intensity scale for that group.

To investigate changes in the phenotype of tumor macrophages following radiation therapy, we FACS sorted tumor macrophages from Panc02 tumors (Figure 2). We have previously shown that we can distinguish mature tumor macrophages from immature myeloid and MDSC populations by expression of Gr1 and IA (MHC class II) [20]. To isolate these sub-populations, we first gated CD11b^+^ cells in the untreated or irradiated tumors (Figure 2A, image i), then sorted the CD11b^+^IA^+^ macrophage population and the CD11b^+^Gr1^hi^ MDSC population (Figure 2A, image ii). Cytospins of the sorted populations demonstrates that the CD11b^+^Gr1^hi^ MDSC predominantly have a granulocyte morphology and the CD11b^+^IA^+^ cells have a macrophage morphology in both the untreated (Figure 2A, image iii) and irradiated tumors (Figure 2A, image iv). RNA was purified from CD11b^+^IA^+^ macrophages from untreated or irradiated tumors 1 day or 7 days following radiation therapy and Gene Expression Microarray analysis was performed. The gene expression pattern confirmed the isolated macrophage phenotype; there was abundant expression of CD14, F4/80, and low or absent expression of B cell, T cell and endothelial markers (Figure 2B). As has previously been described for tissue macrophages [21], tumor macrophages express CD11c [21], [22]. Following radiation therapy expression of CD11c does not change, indicating that there is no change in potential contaminating dendritic cells (CD11c^+^ higher expression) or neutrophils (CD11c^+^ lower expression) and no dramatic changes in inflammatory stimuli in the tumor [23]. Clustering analysis identified 4 major patterns of gene expression. Pattern 1 represented genes that were essentially unchanged between time points, and included the majority of genes. Pattern 2 represented genes that were downregulated at both time points following radiation, pattern 3 represented genes that were upregulated immediately following radiation, but declined on day 7, and pattern 4 represented genes that were upregulated at day 7. These genes are summarized in Figure S2. Analysis of regulated genes (Patterns 2–4) by ontology identified predictable patterns in the regulated genes. Downregulated (pattern 2) genes included those that were related to proliferation and to cell division (Figure S2), and this links closely with genes that were upregulated within a day of radiation (pattern 3), where genes involved in the response to DNA damage, stress and cell death are highly represented. Genes upregulated late involved a range of immune and inflammatory response genes. Since the DNA damage and anti-proliferative effects of radiation therapy were predicted, we examined more closely those genes that would not be predicted to be involved in the direct response to radiation. Following radiation macrophages downregulate a number of genes involved in extracellular matrix development, including *sparc*, a number of collagen genes (*col1a1*, *col1a2, col3a1*, *col6a1*) and biglycan (*bgn*) (Figure S2, image i), suggesting that macrophage support of stroma may be transiently suspended following radiation therapy. Amongst those genes transiently upregulated following radiation, macrophages upregulate *ccl2* and *ccl7* (Figure S2, image ii), each of which have been associated with recruitment of macrophages to infectious and wound sites via the receptor CCR2 [24], [25]. At this day 1 post-radiation time point, there is possible evidence of a pro-inflammatory macrophage response, as indicated by upregulation of *cd80, tnfsf9* (41BBL) and *tnf*, though expression of these genes declines by day 7 post-radiation. Interestingly, at the day 1 time-point, there is upregulation of *mertk* and *gas6*, key genes mediating macrophage phagocytosis of dying cells [26]. By 7 days following radiation, we see upregulation of *ccr2* (Figure S2, image iii), which may represent chemotaxis in response to earlier upregulation of *ccl2* and *ccl7*. In addition, at this time point macrophages upregulate *tlr2* and *tlr4*, potentially indicating heightened responsiveness to endogenous adjuvants released by dying cells. It is of note that *ccr2*, *tlr2* and *tlr4*, along with *cd43*, are the genes causing a high Z-score for the tumor necrosis factor biosynthetic process ontologies at day 7 following radiation (Figure S2, image iii). These changes suggest that significant inflammatory changes in tumor macrophages occur following radiation therapy that may influence the tumor environment.

Despite these fluctuations in gene expression, the tumor macrophages maintain or increase a polarized M2 phenotype, shown by their expression of M1 and M2 macrophage markers (Figure 2C) as defined by Murray and Wynn [22]. Importantly, the tumor macrophages do not express the M1 marker iNos and express substantial levels of the M2 marker arginase I, which significantly increases following radiation therapy (Figure 2C) and has been shown to inhibit T cell function [16], [27]. To validate the microarray data, we FACS sorted CD11b^+^IA^+^ and CD11b^+^Gr1^hi^ populations from Panc02 tumors 7 days following radiation therapy, or from tumors left untreated, and prepared protein lysates from purified cells. Western blotting of these lysates demonstrated increased expression of arginase I in macrophages following radiation therapy, and undetectable expression of iNOS (Figure 2C, image ii). To confirm the lack of iNOS expression in tumor macrophages, we sorted CD11b^+^IA^+^ and CD11b^+^Gr1^hi^ populations from 4T1 tumors and western blotted these lysates alongside lysates of Raw264.7 macrophages cultured alone or with irradiated 4T1 cells (Figure S3). In these experiments, CD11b^+^IA^+^ tumor macrophages, but not CD11b^+^Gr1^hi^ tumor neutrophils express arginase I and neither population expresses detectible iNOS. Importantly, Raw264.7 macrophages decrease their iNOS expression and increase Arginase I expression, indicating a shift from M1 to M2 phenotype. These data indicate while there may be an initial shift towards pro-inflammatory macrophage activation as indicated by TNFα and CD80 upregulation, macrophages within the tumor retain M2 differentiation and that by day 7 following radiation the pro-inflammatory window has resolved. The tumor environment 7 days following radiation may be more suppressive to adaptive immune responses due to the increased number of M2 macrophages with increased expression of arginase I.

**Figure 3:**
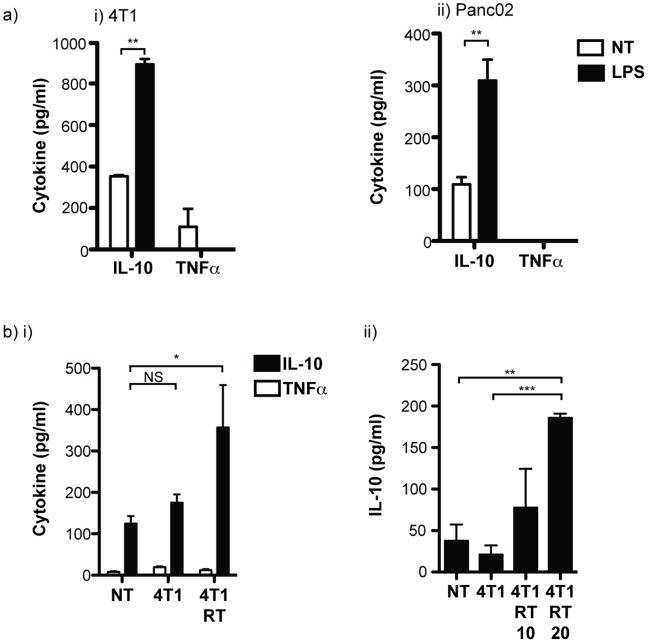
Cytokine responses of tumor macrophages. a) CD11b^+^IA^+^ cells were sorted from i) 4T1 tumors or ii) from Panc02 tumors and treated *in vitro* with 100 ng/ml LPS, or left untreated (NT). 24 hours later supernatants were collected and ELISA tested for secretion of IL-10 and TNFα. b) i) Bone marrow-derived macrophages were incubated alone or with untreated or irradiated 4T1 cancer cells for 24 hours before stimulation with 100 ng/ml LPS. Supernatants were collected and ELISA tested for secretion of IL-10 and TNFα after a further 48 hours. ii) Raw264.7 macrophages were incubated alone or with an equal number of untreated or irradiated 4T1 cancer cells (10 or 20 Gy) for 24 hours, then treated with 100 ng/ml LPS and supernatants collected and ELISA tested for secretion of IL-10 after a further 48 hours. Graphs are representative of multiple replicate experiments.

These data imply that it is these M2-differentiated macrophages that will be exposed to any immunological adjuvants that are released from dying cancer cells following radiation therapy. To evaluate the response of tumor macrophages to adjuvants, we FACS sorted CD11b^+^IA^+^ tumor macrophages from untreated 4T1 or Panc02 tumors and measured their cytokine response to LPS stimulation. Stimulation of purified CD11b^+^IA^+^ cells with LPS causes secretion of IL-10, but not TNFα (Figure 3A). To investigate whether newly recruited macrophages would be polarized in the tumor environment following radiation therapy, we incubated bone marrow-derived macrophages alone, with live 4T1 cancer cells or with irradiated 4T1 cancer cells for 24 hours before stimulation with LPS. Co-culture with irradiated cancer cells increased IL-10 secretion following LPS stimulation (Figure 3B, image i). Similarly, Raw264.7 monocyte/macrophages are only polarized to produce IL-10 following co-culture with irradiated cancer cells (Figure 3B, image ii). These data demonstrate that tumor-associated macrophages are polarized towards an M2 phenotype, and that dying cancer cells generated by radiation therapy may cause M2 polarization of newly recruited macrophages.

**Figure 4:**
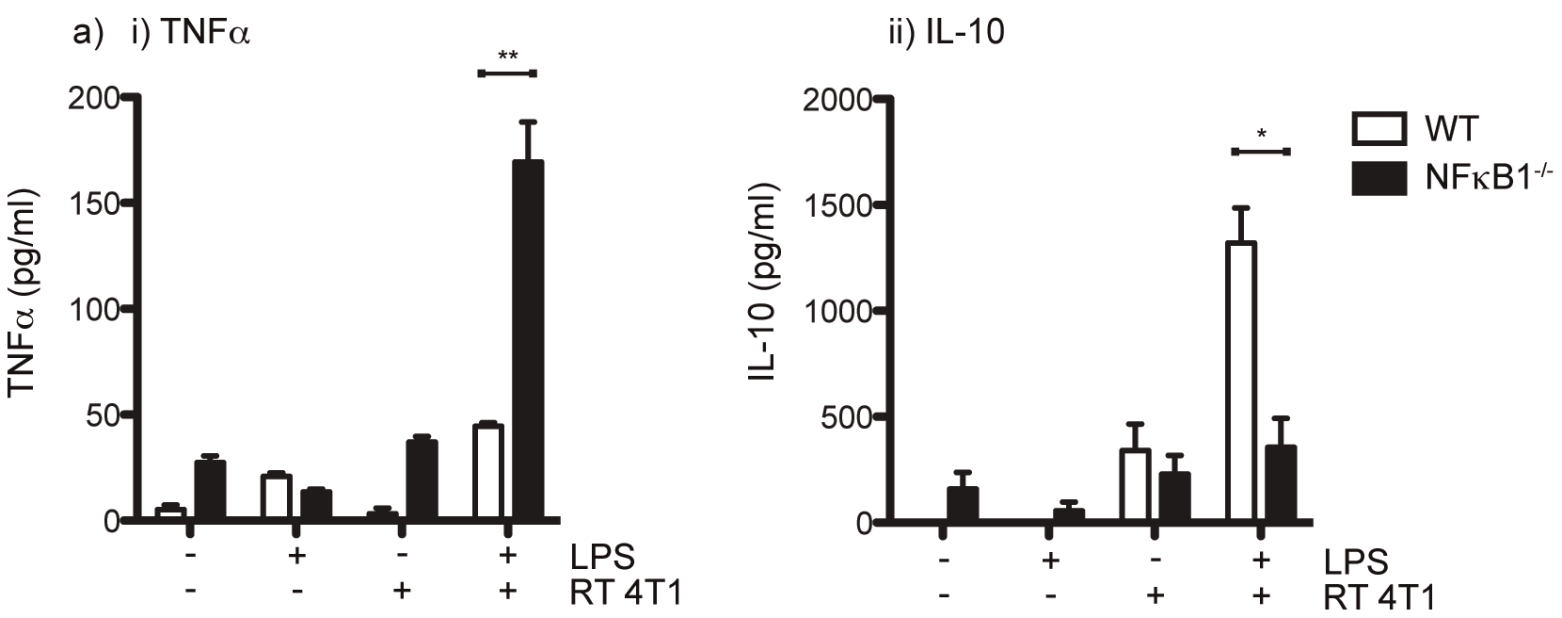
NFκB in macrophage polarization. a) Macrophages were derived from the bone marrow of wild-type (wt) or NFκB1^−/−^ mice and incubated alone or with 10 Gy irradiated 4T1 cancer cells for 24 hours, then treated with 100 ng/ml LPS and ELISA tested for secretion of i) TNFα and ii) IL-10 after a further 48 hours.

Endogenous T cell responses play a significant role in tumor control by radiation therapy, but are generally insufficient to cure tumors in the absence of additional immunotherapy [1], [3], [4]. A number of investigators have characterized a transient improvement in the immune environment of the tumor following radiation therapy [28], [29]; however, our data suggests that tumor macrophages will work against adaptive immune cells by release immunosuppressive cytokines in response to endogenous adjuvants. For these reasons we hypothesized that preventing macrophages from becoming M2 and suppressing adaptive immunity would improve the efficacy of radiation therapy. Classically, pro-inflammatory TNFα gene expression in response to TLR ligation occurs via signal transduction incorporating NFκB heterodimers. To investigate whether NFκB p50 was involved in redirection of the macrophage response, we prepared bone marrow macrophages from wild-type mice or from NFκB1 knockout mice that are deleted for the p105 precursor protein of NFκB p50. We demonstrate that in the presence of irradiated 4T1 cells, bone marrow macrophages from wild-type mice secrete IL-10 in response to LPS stimulation, while NFκB1 knockout mice secrete TNFα (Figure 4). As seen in purified tumor macrophages (Figure 3A) cytokine production following co-culture is dependent on LPS stimulation, indicating that dying cells provide a differentiation signal with macrophage polarization becoming evident following stimulation. These data demonstrate macrophage deviation to IL-10 production requires the transcriptional activity of NFκB p50.

**Figure 5:**
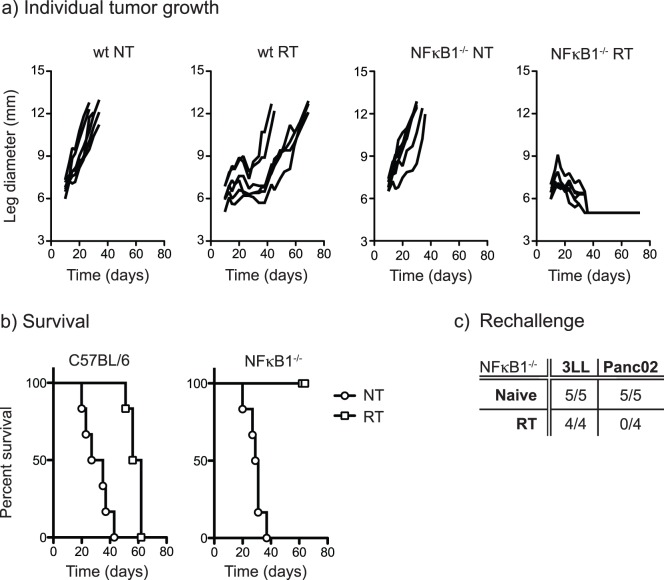
Radiation therapy of tumors in NFκB1 knockout mice. a) Wild-type (wt) or NFκB1^−/−^ C57BL/6 mice were challenged with 2×10^5^ Panc02 *s.c.* in the right leg and mice received 3 daily doses of 20 Gy focal radiation to the leg beginning on day 14 (RT) or were left untreated (NT).Tumor leg diameter was measured 3× per week. b) Survival curves of mice in a replicate experiment treated as in a) where mice were euthanized at a leg diameter exceeding 12 mm. c) Proportion of tumors growing following s.c. injection of 3LL or Panc02 in NFκB1^−/−^ naïve mice or NFκB1^−/−^ mice that were cured of their primary tumor with radiation therapy (RT).

To determine the consequence of redirected macrophage polarization *in vivo*, we established Panc02 tumors in wild-type or NFκB1 knockout mice and treated these mice with radiation therapy. Despite radiation therapy, all tumors eventually recur in wild-type mice (Figure 5A). However, in NFκB1 knockout mice, tumors are controlled by radiation. Survival in mice bearing untreated tumors is not different between wild-type or NFκB1 knockout mice, but is significantly different following radiation therapy (wtNT *vs.* wtRT p<0.001; wtNT *vs.* NFκB1^−/−^NT NS; NFκB1^−/−^NT *vs.* NFκB1^−/−^RT p<0.005; wtRT *vs.* NFκB1^−/−^RT p<0.001) (Figure 5B). To determine whether tumor treatment resulted in endogenous immune protection, surviving mice were rechallenged with Panc02 or irrelevant 3LL tumors. Both Panc02 and 3LL grew in naïve NFκB1^−/−^ mice. NFκB1^−/−^ mice that were cleared of their primary tumor by radiation therapy were protected against rechallenge with Panc02 tumors, but remained susceptible to 3LL tumors (Figure 5C). These data demonstrate that mice bearing Panc02 tumors that were cleared by radiation therapy also developed an endogenous tumor antigen-specific response and long-term protective immunity to the primary tumor. Together, these data support the hypothesis that tumor macrophage polarization limits the efficacy of radiation therapy, and demonstrate that expression of NFκB1 limits the efficacy of radiation therapy *in vivo*.

## Discussion

These data demonstrate that tumors can recur despite significant reductions in the clonogenic potential of cancer cells following hypofractionated radiation therapy (Figure 1). Treatment failure is associated with an influx of macrophages into the tumor site (Figure 1) that exhibit an immune suppressive and M2 phenotype by 7 days post radiation treatment (Figure 2). We demonstrate that adjuvant causes M2 polarized macrophages in the tumor to secrete the immune suppressive cytokine IL-10 (Figure 3), and that that polarization of macrophages by dying cancer cells occurs through a transcriptional switch via regulation of NFκB p50 (Figure 4). The consequence is that radiation therapy is more effective in mice deficient in NFκB p50 (Figure 5). These data demonstrate that the established tumor environment is not optimal for adaptive immunity, and that while cytotoxic therapy may release tumor antigens and endogenous adjuvants, the consequence may be further antigen-specific immune suppression at the tumor site. It is important to note that while the untreated tumor has M2 differentiated macrophages, immediately following radiation therapy there may be a transient upregulation of M1 signals that resolves as the tumor transitions to repair. However, while the macrophages recruited to the tumor following radiation therapy would not be expected to be pre-polarized to either M1 or M2 phenotypes, they enter an environment already populated with M2 macrophages and on entry the newly recruited macrophages are able to interact with dying cells. That the expression of M2 markers is sustained or increases following radiation therapy suggests that the irradiated tumor environment drives M2 differentiation of these newly recruited cells. The result is M2 differentiation of both new and existing tumor macrophages. Radiation alone rarely results in tumor-specific immunity capable of destroying untargeted tumors – the elusive abscopal effect. However, immunotherapy in combination with radiation therapy have shown abscopal activity in animal models [4]. It is possible that well-timed T cell targeted immune interventions can overcome the negative environment, for example by increased infiltration of the tumor by T cells that produce inflammatory cytokines [20]. The experiments described here suggest that alternative therapeutic interventions targeting macrophage differentiation will be complimentary and can be exploited to optimize adaptive immune control of residual disease.

These data have implications for the integration of radiation therapy and immunotherapies in the clinic. While many immunotherapies are effective in animal models, their success commonly depends on treatment very early in tumor development. In transplantable tumor models it takes at least 10 days to establish a suppressive environment made up of suppressive macrophages and regulatory T cells [30], [31]. Potent immunotherapeutic antibodies are no longer effective if tumors have time to establish their immune suppressive environment beyond 10 days [20], [32], [33]. This scenario is relevant for treatment of cancer patients since tumors accumulate an array of immune regulatory features as part of their evolution into clinically relevant malignancies. Despite the increased immune suppression that we describe following radiation, there may be a transient window of classical inflammation in the tumor before inflammatory resolution is established and the tumor is repaired. Over the course of radiation, patients have been shown to develop tumor antigen-specific immune responses that were not detectable before treatment [34]. In addition, in animal models, radiation therapy is less effective if endogenous CD8 T cell responses are eliminated [1], [3]. Radiation has been shown to render the tumor site transiently more attractive to effector T cells [28], [29] and the combination of radiation with multiple infusions of tumor-specific effector T cells can combine to alter the wound repair phenotype of the tumor providing an extended mechanism of tumor control [35]. Thus, in the face of an established, suppressive tumor it may be necessary to use multiple aggressive therapies for effective immune control of residual disease.

The specific roles of the classical M1 cytokine TNFα as opposed to the M2 cytokine IL-10 response is not clear; each cytokine may simply be an indicator of broader macrophage differentiation. However, TNFα has been shown to inhibit tumor angiogenesis [36], synergize with radiation therapy [37], and a spike in serum TNFα was associated with one documented case of abscopal tumor regression [38]. By contrast, IL-10 feeds back on macrophages to increase alternative macrophage differentiation [39], and the progressive induction of IL-10 in tumor infiltrating cells during tumor growth has been shown to suppress anti-tumor adaptive immune responses [40]. IL-10 can be effectively blocked with specific antibodies to the cytokine and to the IL-10 receptor, and blockade of IL-10 has been shown to result in more effective immune control of tumors [41].

The experiments described here identify tumor macrophages as potential therapeutic targets to increase immune control of residual disease following radiation therapy. In animal models, local control by radiation therapy has been improved by preventing macrophage influx following radiation by total body irradiation [42] or by antibodies specific to Mac-1 [43]. Rather than depleting macrophages, it has also been proposed that interfering with the polarization of tumor macrophages is a potential therapeutic strategy [44]. While this study focuses on the contribution of macrophage polarization to tumor control by radiation therapy, the ubiquity of NFκB in transcriptional regulation means that other cell types may play a role in the outcome of the *in vivo* radiation therapy experiments. Ongoing work in the laboratory aims to identify he contribution of other cells, such as T cells and endothelial cells in NFκB1 deficient mice.

The accumulation of NFκB p50 and the formation of transcriptionally regulatory p50 homodimers appear to play an important role in the resolution of inflammation. While clearance of bacterial infections is not altered by the absence of NFκB1, NFκB p50 deficient mice exhibit excessive and prolonged inflammation following bacterial clearance [45], and the consequence of inflammatory injuries is more severe in the absence of NFκB p50 [46]. Saccani *et al.* demonstrated that macrophages isolated from tumors have increased expression of NFκB p50, and accumulate NFκB p50 homodimers in the nucleus [47]. NFκB p50 homodimers can bind to the same NFκB sites in the TNFα promoter as conventional NFκB heterodimers, but result in transcriptional inhibition rather than transcriptional activation [48]. By contrast, the same complexes promote transcription of IL-10 [48]. Our data showing that NFκB p50 deficient macrophages sustain M1 differentiation even in the presence of irradiated cancer cells is consistent with data from Saccani *et al.* with M2 tumor macrophages [47], and interestingly also with data from Porta *et al.* with tolerized macrophages [49]. It is interesting that tolerized macrophages closely resemble M2 macrophages [49], [50], [51], and it is possible that M1 and M2 differentiation may be temporally regulated in addition to regulation via inflammatory mediators and regulatory cytokines [52]. Our data fits a model where the tumor environment post-cytotoxic therapy is a site of inflammatory resolution, and we propose that this process of inflammatory resolution is part of an endogenous process of repair that can protect the tumor site for eventual recurrence. In the response to infections or following administration of sterile irritants, preventing immune resolution results in increased pathology. Thus, we propose that sustaining M1 differentiation, or creating a non-resolving environment in the tumor will increase destruction at the inflammatory site. In cancer therapy, increased destruction at a *focused* target site could be considered a benefit.

## Supporting Information

Figure S1 **Margin to margin overview of tumor histology following radiation therapy.** C57BL/6 mice were challenged with 2×10^5^ Panc02 *s.c.* in the right leg and mice received 3 daily doses of 20 Gy focal radiation to the leg beginning on day 14 (RT) or were left untreated (NT). Tumors were harvested for histology 7 days following the final radiation dose. Images show neighboring sections from tumors receiving NT or RT that were H&E stained or underwent immunofluorescence staining with antibodies specific for VWF and F4/80, and detected with antibodies conjugated to AF488 (Green) and AF568 (Red), respectively. Nuclear material was counterstained with DAPI (Blue) and sections were imaged by confocal microscopy. Multiple digital images were taken from the tumor margin to the opposite margin and digitally stitched to recreate a margin-to-margin overview of a representative tumor.
(TIF)

Figure S2 **Cluster analysis was performed using Genesifter software to identify four patterns of gene expression.** Gene expression Pattern 1 was little changed between samples. Within gene expression Pattern i) 2, ii) 3, and iii) 4, only those genes characterized as present and that demonstrate significant differences in gene expression (ANOVA) were included in ontology analysis (top). Groups are sorted by Z-score. Gene lists within these clusters (bottom) are limited to those showing significant differences in gene expression (ANOVA) and greater than 1.5 fold changes in gene expression. Final gene lists are sorted by peak expression and the top 80 genes are shown (where sufficient numbers matching these criteria are present), separated into groups of 20 genes per column with an individual key showing the gene intensity scale for that group.
(TIF)

Figure S3 **Macrophage polarization from iNOS to Arginase expression by irradiated cancer cells** a) Tumor infiltrating cells from 4T1 tumors were harvested and i) gated CD11b^+^ cells were FACS sorted according to expression of Gr1 and IA. Sorted populations of ii) CD11b^+^Gr1^hi^ and iii) CD11b^+^IA^+^ cells were used to prepare protein lystates. a) Western blot of protein lysates from sorted tumor myeloid cells (lanes 3 and 4) alongside lysates from Raw264.7 macrophages incubated alone or with equal numbers of irradiated 4T1 cells (lanes 1 and 2). Lanes were loaded with equal protein and probed with antibodies specific for Arginase I, iNOS and GAPdH. The image represents 3 western blots cropped and positioned above each other to show detection of Arginase I, iNos and GAPDH.
(TIF)
