# Supplementary figures and images for: Expression of NF-κB p50 in Tumor Stroma Limits the Control of Tumors by Radiation Therapy
Source: PLoS One. 2012 Jun 28;7(6):e39295. doi: 10.1371/journal.pone.0039295 (PMC3386283; doi:10.1371/journal.pone.0039295)

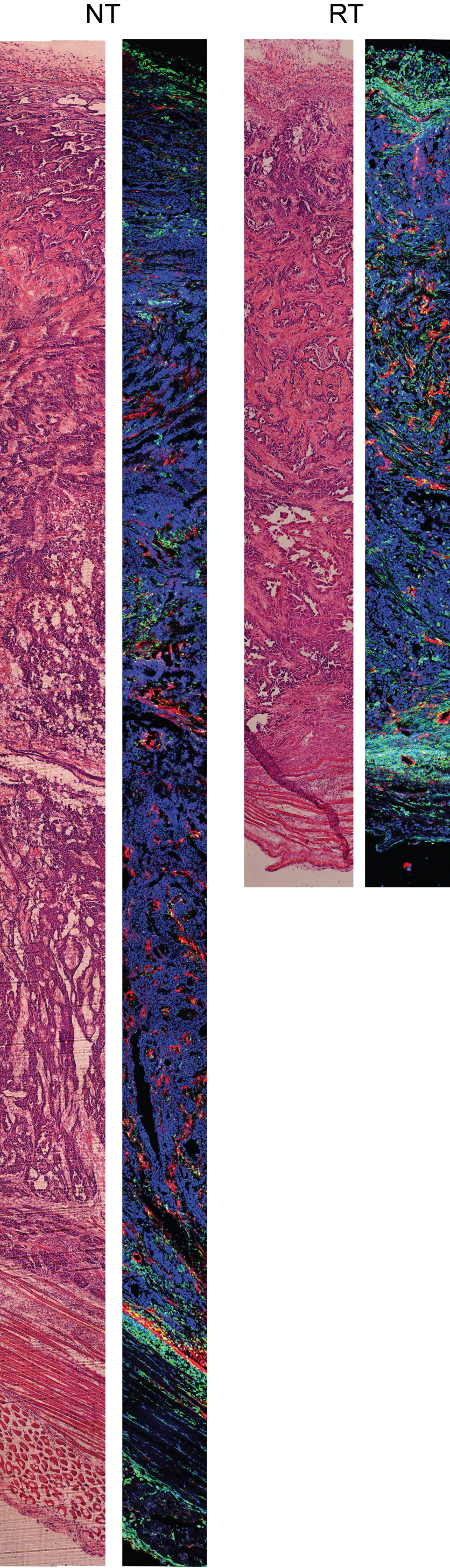

Supplement: Figure S1 — Margin to margin overview of tumor histology following radiation therapy. C57BL/6 mice were challenged with 2×10^5^ Panc02 s.c. in the right leg and mice received 3 daily doses of 20 Gy focal radiation to the leg beginning on day 14 (RT) or were left untreated (NT). Tumors were harvested for histology 7 days following the final radiation dose. Images show neighboring sections from tumors receiving NT or RT that were H&E stained or underwent immunofluorescence staining with antibodies specific for VWF and F4/80, and detected with antibodies conjugated to AF488 (Green) and AF568 (Red), respectively. Nuclear material was counterstained with DAPI (Blue) and sections were imaged by confocal microscopy. Multiple digital images were taken from the tumor margin to the opposite margin and digitally stitched to recreate a margin-to-margin overview of a representative tumor. (TIF) [file pone.0039295.s001.tif]

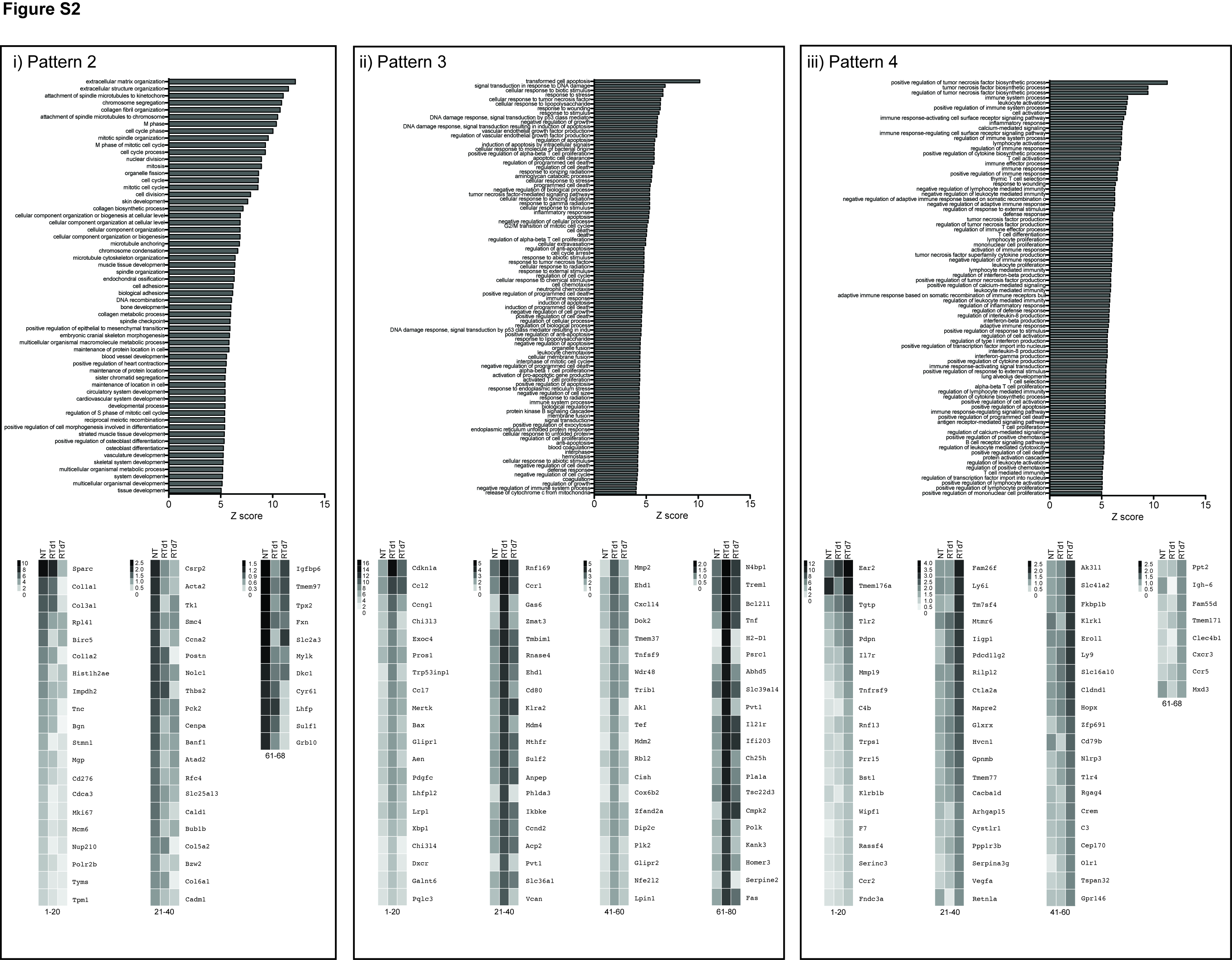

Supplement: Figure S2 — Cluster analysis was performed using Genesifter software to identify four patterns of gene expression. Gene expression Pattern 1 was little changed between samples. Within gene expression Pattern i) 2, ii) 3, and iii) 4, only those genes characterized as present and that demonstrate significant differences in gene expression (ANOVA) were included in ontology analysis (top). Groups are sorted by Z-score. Gene lists within these clusters (bottom) are limited to those showing significant differences in gene expression (ANOVA) and greater than 1.5 fold changes in gene expression. Final gene lists are sorted by peak expression and the top 80 genes are shown (where sufficient numbers matching these criteria are present), separated into groups of 20 genes per column with an individual key showing the gene intensity scale for that group. (TIF) [file pone.0039295.s002.tif]

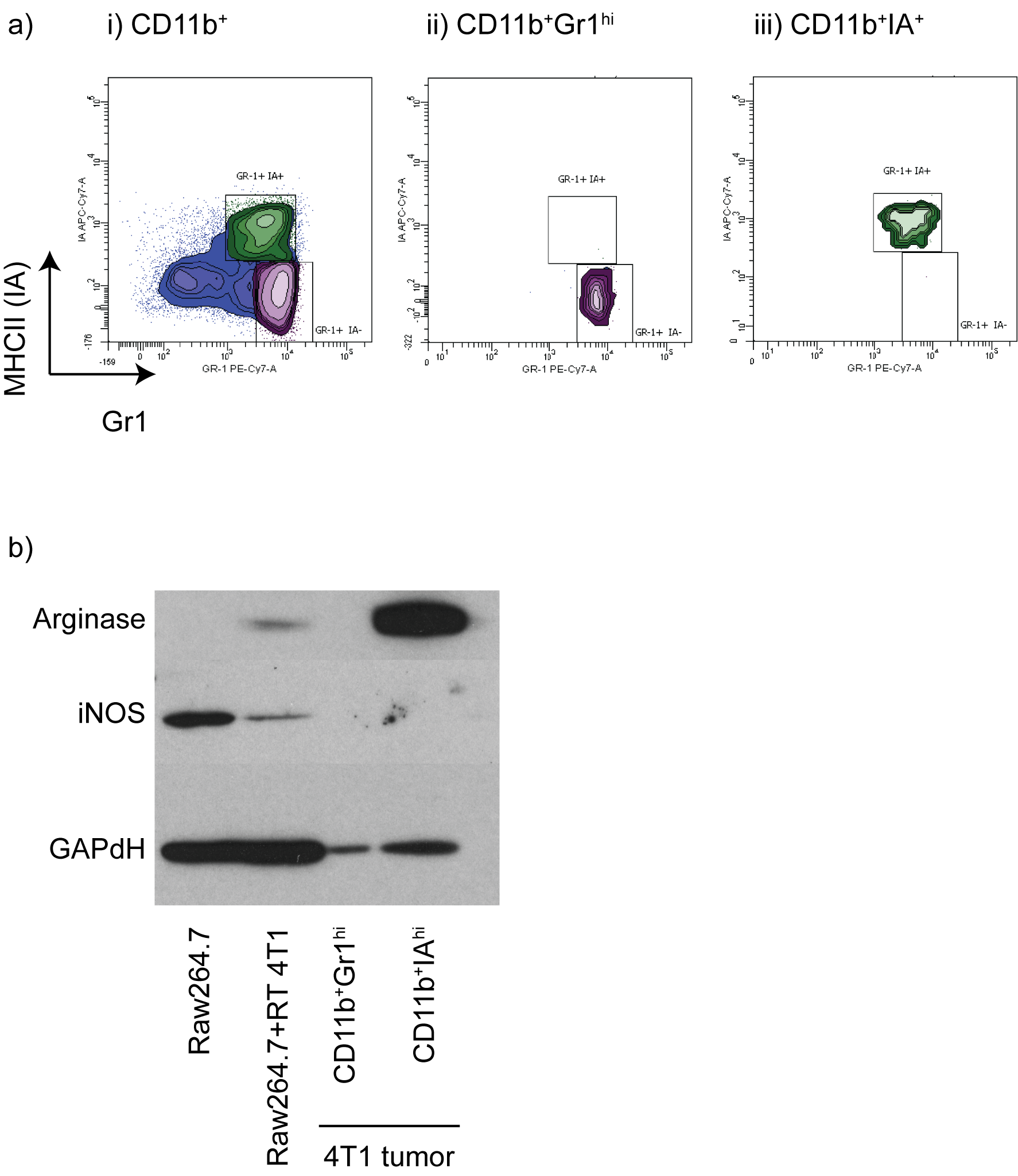

Supplement: Figure S3 — Macrophage polarization from iNOS to Arginase expression by irradiated cancer cells a) Tumor infiltrating cells from 4T1 tumors were harvested and i) gated CD11b+ cells were FACS sorted according to expression of Gr1 and IA. Sorted populations of ii) CD11b+Gr1hi and iii) CD11b+IA+ cells were used to prepare protein lystates. a) Western blot of protein lysates from sorted tumor myeloid cells (lanes 3 and 4) alongside lysates from Raw264.7 macrophages incubated alone or with equal numbers of irradiated 4T1 cells (lanes 1 and 2). Lanes were loaded with equal protein and probed with antibodies specific for Arginase I, iNOS and GAPdH. The image represents 3 western blots cropped and positioned above each other to show detection of Arginase I, iNos and GAPDH. (TIF) [file pone.0039295.s003.tif]
